# Stress-induced tunneling nanotube communication in CNS glial cells: implications for inflammatory demyelination and repair failure

**DOI:** 10.3389/fncel.2026.1949981

**Published:** 2026-09-15

**Authors:** Yanjun Liu, Mingming Li, Wei Liu, Jia Guo, Jian Yin

**Affiliations:** 1 Department of Neurology, The Second Hospital of Lanzhou University, Lanzhou, China; 2 Department of Neurology, National Center for Gerontology, Beijing Hospital, Beijing, China

**Keywords:** inflammatory demyelinating disorders, mitochondrial transfer, multiple sclerosis, neuroimmunology, remyelination failure, tunneling nanotubes

## Abstract

Multiple sclerosis and related inflammatory demyelinating disorders of the central nervous system, including neuromyelitis optica spectrum disorder and myelin oligodendrocyte glycoprotein antibody-associated disease, are characterized by immune-mediated tissue injury, glial dysfunction, metabolic stress, and incomplete repair. Although peripheral immune infiltration and antibody-mediated mechanisms explain many aspects of acute lesion formation, they do not fully account for persistent lesion activity, metabolic instability, or remyelination failure within the local tissue microenvironment. Tunneling nanotubes are thin, actin-rich membranous connections that enable direct intercellular transfer of organelles, vesicles, proteins, ions, and stress-related cargoes. In the nervous system, tunneling nanotube-mediated communication has been implicated in mitochondrial transfer, glial-neuronal interactions, pathological cargo dissemination, and cellular stress adaptation. This review examines the potential role of tunneling nanotubes in multiple sclerosis and related inflammatory demyelinating disorders from a lesion-centered perspective. Moving beyond general descriptions of TNT biology, glial communication, and mitochondrial transfer, we consider how TNT-mediated intercellular exchange may intersect with pathological cargo transfer, metabolic stress and support, neurovascular dysfunction, and impaired repair within demyelinating lesions. In multiple sclerosis, they may be particularly relevant to chronic active lesion edges, glial stress responses, and remyelination failure. In aquaporin-4-IgG-positive neuromyelitis optica spectrum disorder, they may be more closely related to astrocytic injury and neurovascular-unit stress. In myelin oligodendrocyte glycoprotein antibody-associated disease, their potential relevance is more speculative and may lie in post-inflammatory repair coordination. Direct evidence remains limited. Future studies should prioritize rigorous *in situ* identification, cell-type-specific analysis, cargo tracking, and causal intervention experiments before tunneling nanotubes can be considered a validated disease mechanism.

## 1 Introduction

Multiple sclerosis and related inflammatory demyelinating disorders of the central nervous system comprise a heterogeneous group of diseases characterized by immune-mediated injury to myelin, glial cells, and the neurovascular unit. These types of diseases are characterized by myelin loss with relative preservation of axons, occurring against a backdrop of inflammation. The common pathology is marked by neuroinflammation, myelin loss, and reactive astrogliosis (Allegretta et al., 2022). This category of diseases encompasses a wide spectrum of clinical conditions, primarily including multiple sclerosis (MS), neuromyelitis optica spectrum disorders (NMOSDs), and myelin oligodendrocyte glycoprotein antibody-associated disease (MOGADs) (Allegretta et al., 2022; Qiu et al., 2019). Clinically, these disorders may present with acute inflammatory attacks, demyelinating lesions, and variable neurological deficits, including visual impairment, motor dysfunction, sensory disturbance, ataxia, and cognitive changes. An important premise of this review is that these entities share inflammatory demyelination but differ in dominant target cells, lesion biology, and repair potential. They should therefore not be regarded as different manifestations of a single pathological process. MS is characterized by immune-mediated demyelination and chronic lesion evolution. In contrast, AQP4-IgG-positive NMOSD is primarily an autoimmune astrocytopathy, whereas MOGAD is an antibody-associated inflammatory demyelinating disorder directed against myelin oligodendrocyte glycoprotein.

Over recent decades, substantial progress has been made in defining the immune mechanisms underlying these disorders. Nevertheless, lesion progression cannot be fully attributed to peripheral immune infiltration or soluble inflammatory mediators. Significant heterogeneity among glial and immune cells has been observed at the edges of chronic active demyelinating white matter lesions, including distinct phenotypes such as MS inflammatory microglia and MS inflammatory astrocytes, which show features associated with neurodegenerative programming (Absinta et al., 2021). In many lesions, persistence and progression appear to depend not only on immune activity but also on local interactions among microglia, astrocytes, oligodendrocyte-lineage cells, neurons, and vascular-associated cells. These interactions influence lesion expansion, axonal vulnerability, metabolic imbalance, and remyelination failure. In MS, blood-brain barrier dysfunction may facilitate immune-cell infiltration and activate resident microglia and astrocytes. Microglia, astrocytes, and oligodendrocyte-lineage cells then act as important mediators of neuroinflammation and tissue remodeling (Chu et al., 2021). However, the intercellular mechanisms that organize these local responses within the lesion microenvironment remain incompletely understood.

Intercellular communication plays a critical role in maintaining homeostasis and regulating immune responses within the central nervous system (CNS) and operates through a variety of pathways (Potokar et al., 2026). Previous studies on intercellular communication in neuroinflammatory and demyelinating disorders have mainly focused on cytokine and chemokine networks, receptor-ligand interactions, gap junctions, extracellular vesicles, and metabolic coupling. Unlike these routes, TNTs provide direct physical connections between defined cell pairs and can support the transfer of relatively large intracellular cargoes, including intact organelles. TNTs are of particular interest because they provide direct physical continuity between defined cell pairs and permit transfer of cargoes, including intact organelles, that cannot readily pass through conventional channel- or diffusion-based communication routes. Compared with these better-studied pathways, tunneling nanotubes (TNTs) represent a more direct form of cell-to-cell communication that remains insufficiently explored in MS and related inflammatory demyelinating disorders. TNTs are slender membranous projections that connect the cytoplasmic compartments of adjacent or distant cells and permit the transfer of diverse cargoes. They can mediate directional transport of intracellular components, including vesicles, proteins, ion signals, and mitochondria. Microglial TNTs have also been shown to transfer mitochondrial particles between cells (Chakraborty et al., 2023a).

In the CNS, TNT-mediated communication has been associated with mitochondrial transfer, pathological protein propagation, stress adaptation, and glial–neuronal interactions. These functions are relevant to inflammatory demyelinating lesions, where oxidative injury, mitochondrial dysfunction, metabolic stress, barrier disruption, and dynamic glial responses coexist. TNTs therefore provide a potential route through which local cells redistribute stress signals and metabolic resources and influence tissue repair. This may be particularly relevant in chronic lesions, where overt inflammation may decline while tissue injury and remyelination failure persist.

Recent reviews have summarized TNT biology in the nervous system, glial communication, astrocyte-neuron crosstalk, and TNT-mediated mitochondrial transfer. Here, mitochondrial transfer is considered one component of a broader stress-adaptive and pathological cargo-exchange network. Rather than providing another general overview of TNT structure or cargo transfer, this review examines TNT communication within the lesion biology of inflammatory demyelinating disorders. We propose a dynamic model in which inflammatory and oxidative stress promote mitochondrial dysfunction and cytoskeletal remodeling, thereby influencing TNT formation and cargo exchange. Depending on the cargo and the state of donor and recipient cells, TNT-mediated communication may either support cellular adaptation and repair or reinforce local injury and lesion persistence. We further distinguish direct disease-related evidence from mechanistic extrapolation and define the experimental requirements needed to establish TNTs as a causal mechanism in demyelinating lesions.

## 2 Methods

### 2.1 Literature search strategy

This article was designed as a narrative and critical review rather than a systematic review or meta-analysis. A structured literature search was performed to identify publications relevant to tunneling nanotubes (TNTs), central nervous system intercellular communication, mitochondrial and organelle transfer, glial stress responses, inflammatory demyelination, neurovascular injury, and repair failure. Searches were conducted in PubMed/MEDLINE, Web of Science Core Collection, Scopus, and Google Scholar from 1 Jan 2000 to 30 April 2026. The reference lists of selected articles were also manually screened to identify additional relevant studies.

The search strategy was based on four predefined keyword domains. Because the abbreviation “TNT” is non-specific, it was used only in combination with more specific terms such as “tunneling nanotube,” “cell-cell communication,” “mitochondrial transfer,” or “intercellular nanotube.”

The second domain focused on intercellular transfer and cellular cargoes and included “mitochondrial transfer,” “organelle transfer,” “cargo transfer,” “vesicle transfer,” “lysosome,” “endosome,” “pathological protein transfer,” “aggregate transfer,” “cell-to-cell communication,” and “intercellular communication.”

The third domain focused on central nervous system cell types and lesion-relevant structures and included “central nervous system,” “astrocyte,” “microglia,” “oligodendrocyte,” “oligodendrocyte precursor cell,” “neuron,” “axon,” “glia,” “blood-brain barrier,” “neurovascular unit,” “endothelial cell,” and “pericyte.”

The fourth domain focused on inflammatory demyelinating disorders and lesion biology and included “multiple sclerosis,” “MS,” “demyelination,” “remyelination,” “chronic active lesion,” “lesion edge,” “remyelination failure,” “neuromyelitis optica spectrum disorder,” “NMOSD,” “aquaporin-4,” “AQP4,” “myelin oligodendrocyte glycoprotein antibody-associated disease,” “MOGAD,” and “MOG-IgG.”

Representative search strings included the following:

(“tunneling nanotube” OR “tunneling nanotube” OR “membrane nanotube” OR “intercellular nanotube” OR “nanotubular bridge”) AND (“multiple sclerosis” OR “demyelination” OR “remyelination” OR “chronic active lesion” OR “smoldering lesion”).

(“tunneling nanotube” OR “tunneling nanotube” OR “membrane nanotube” OR “intercellular nanotube”) AND (“astrocyte” OR “microglia” OR “oligodendrocyte” OR “neuron” OR “glia” OR “central nervous system”).

(“tunneling nanotube” OR “tunneling nanotube”) AND (“mitochondrial transfer” OR “organelle transfer” OR “cargo transfer” OR “oxidative stress” OR “inflammation”).

(“tunneling nanotube” OR “tunneling nanotube”) AND (“blood-brain barrier” OR “neurovascular unit” OR “endothelial cell” OR “pericyte”).

(“multiple sclerosis” OR “NMOSD” OR “MOGAD” OR “AQP4” OR “MOG-IgG”) AND (“mitochondrial dysfunction” OR “metabolic support” OR “remyelination failure” OR “neurovascular unit” OR “chronic active lesion”).

### 2.2 Selection of literature

The literature search and initial title and abstract screening were performed by one reviewer. The final set of included articles was independently checked by a second reviewer, and any disagreements regarding eligibility were resolved through discussion and consensus. Original experimental studies, human pathological or clinical studies, mechanistic studies, and high-quality review articles were considered. Priority was given to studies that directly examined TNTs or TNT-like structures, studies involving central nervous system cell types, and studies relevant to inflammatory demyelination, glial stress, mitochondrial dysfunction, neurovascular injury, or remyelination. Studies outside the central nervous system were included only when they provided mechanistic information relevant to TNT formation, cargo transfer, inflammatory regulation, or methodological identification.

Articles were excluded if they were not directly relevant to TNT biology or inflammatory demyelinating disease mechanisms, if they used the abbreviation “TNT” for unrelated concepts, if they lacked sufficient mechanistic or methodological detail, or if they were conference abstracts, commentaries, or non-peer-reviewed materials without original data or substantial critical synthesis. Only English-language publications were included.

Because this study was designed as a narrative and critical review rather than a systematic review, the numbers of records initially retrieved, deduplicated, and screened were not prospectively recorded and were therefore not retrospectively estimated. A total of 36 publications meeting the predefined scope and relevance criteria were included in the final narrative synthesis.

### 2.3 Critical appraisal of evidence

All included publications were evaluated using four predefined criteria. First, we assessed the directness of evidence. Studies examining TNTs or TNT-mediated cargo transfer in disease-relevant tissues, demyelinating models, or lesion-associated cell types were considered more direct, whereas studies in unrelated cell systems or non-demyelinating models were treated as indirect mechanistic evidence. Second, we assessed the rigor of TNT identification. Because TNTs lack a specific molecular marker, stronger evidence was assigned to studies combining multiple structural and functional features, including thin F-actin-rich intercellular bridges, membrane and, where shown, cytoplasmic continuity, three-dimensional imaging demonstrating substrate-detached structures, live-cell imaging of TNT formation and dynamics, and direct cargo tracking. We also considered whether TNT-like structures were adequately distinguished from filopodia, cytonemes, retraction fibers, and other membrane protrusions by morphology, substrate attachment, connectivity, dynamics, and cargo-transfer capacity. Studies based only on static images were interpreted cautiously.

Third, we assessed cell-type and disease relevance. Evidence involving astrocytes, microglia, oligodendrocyte-lineage cells, neurons, endothelial cells, pericytes, or blood-brain barrier-associated cells was considered more relevant than evidence from unrelated peripheral or tumor systems. MS, NMOSD, and MOGAD were evaluated separately because they differ in primary cellular targets, lesion pathology, neurovascular involvement, and repair potential. Fourth, we assessed functional interpretation, including links between TNT-mediated communication and pathological cargo dissemination, mitochondrial or metabolic support, inflammatory signaling, neurovascular stress, remyelination failure, or tissue repair. Evidence combining cargo transfer with functional consequences was considered stronger than evidence showing TNT-like structures alone. Major limitations were evaluated according to model relevance, rigor of TNT identification, direct demonstration of cargo transfer and functional effects, and validation in disease-relevant tissues or *in vivo* models. Evidence from neurodegenerative, ischemic, cancer, or peripheral-cell systems was interpreted cautiously when extrapolated to inflammatory demyelinating disorders.

Based on this appraisal, evidence was categorized as direct, indirect, low, or very limited. Direct evidence referred to TNTs or TNT-mediated cargo transfer demonstrated in disease-relevant demyelinating lesions or models. Indirect evidence referred to TNT biology demonstrated in CNS cell types or related neurological injury models but not directly in inflammatory demyelinating disease. Low or very limited evidence referred to disease associations, biological plausibility, or mechanistic extrapolations without direct lesion-based validation. These tiers were used throughout the review to distinguish established observations from hypothesis-generating interpretations. Findings from human inflammatory demyelinating lesions or disease-relevant demyelinating models were considered direct evidence, whereas observations from neurodegenerative, ischemic, cancer, or peripheral-cell models were treated as indirect mechanistic evidence rather than proof of disease-specific involvement. Table 1 summarizes the key experimental studies, including the experimental model, cell type or interaction, TNT-identification approach, and transferred cargo. Functional interpretation, evidence strength, and major limitations are considered in the critical appraisal above, whereas Table 2 provides a complementary disease-level synthesis of predicted cellular interactions, candidate cargoes, proposed functions, evidence levels, and unresolved questions.

**TABLE 1:** Summary and critical appraisal of key experimental evidence for TNT-mediated communication relevant to inflammatory demyelinating disorders.

References	Experimental model	Cell type/interaction	TNT-identification method	Cargo
Chakraborty et al., 2023a; Dias et al., 2026	Human HMC3 microglial and SH-SY5Y neuronal cell co-culture with α-synuclein fibril loading	Microglia–microglia; neuron–microglia	Membrane and F-actin staining; optical Z-stack analysis demonstrating substrate-detached connections; confocal microscopy and 3D reconstruction; live-cell cargo tracking	α-synuclein aggregates; mitochondria
Wang et al., 2011	H_2_O_2_- or serum-deprivation-induced stress in primary rat hippocampal astrocytes and neurons	Astrocyte–astrocyte; astrocyte–neuron	Morphological identification of substrate-detached membrane bridges; F-actin and plasma-membrane labeling; confocal Z-axis analysis; actin-disruption experiments; fluorescent cargo tracking	Mitochondria, endoplasmic reticulum, Golgi, endosomes, intracellular and extracellular Aβ
Baghaie et al., 2026	PANC-1 pancreatic, MCF-7 breast, and SW620 colorectal cancer cells exposed to TGF-β1, IL-6, or HGF	Cancer cell–cancer cell	Fluorescence microscopy with quantitative image analysis of TNT-like conduits	Not directly assessed
Goodman et al., 2019	Murine bone marrow-derived or IC-21 macrophages co-cultured with cystinotic fibroblasts; macrophage polarization with LPS/IFNγ or IL-4/IL-10	Macrophage–fibroblast	Actin/tubulin immunostaining; confocal Z-stacks and 3D reconstruction showing protrusions above the substratum; cytochalasin sensitivity; fluorescent organelle tracking	Cystinosin-bearing lysosomes; mitochondria
Chen et al., 2025	Adipose-derived stem cell–macrophage communication in gestational diabetes mellitus models	Adipose-derived stem cells–adipose tissue macrophages	Live-cell imaging, scanning electron microscopy, and mitochondrial tracking; mechanistic interrogation of the WNT5A–RhoA–ROCK1 axis	Mitochondria
Pisani et al., 2022; Rostami et al., 2021	Human BBB cell co-cultures and 3D BBB assembloids; astrocytic OGD/reoxygenation injury	Pericyte–astrocyte; endothelial cell–astrocyte	Live time-lapse microscopy; F-actin labeling; confocal Z-stack and 3D reconstruction demonstrating substrate-detached TNTs; mitochondrial tracking; cytoskeletal perturbation	Functional mitochondria
Rostami et al., 2021; Scheiblich et al., 2024	Human iPSC-derived astrocyte–microglia co-cultures exposed to α-synuclein or Aβ fibrils	Astrocyte–microglia	Time-lapse live-cell microscopy; plasma-membrane labeling; visualization of nanotubular and other direct membrane contacts; intracellular aggregate tracking	α-synuclein and Aβ aggregates
Scheiblich et al., 2024; Sun et al., 2026	Primary cortical neuron–microglia co-culture with α-synuclein or tau aggregate loading	Microglia–neuron	Electron microscopy; membrane/F-actin staining; super-resolution 3D confocal Z-stack imaging of free-floating TNTs; live time-lapse cargo tracking; cytoskeletal/signaling perturbation	α-synuclein and tau aggregates; mitochondria

Direct evidence was defined as demonstration of TNTs or TNT-mediated cargo transfer in human inflammatory demyelinating lesions or disease-relevant demyelinating models. No such direct evidence was identified among the studies included in this review; therefore, the studies summarized here provide indirect mechanistic evidence from CNS stress, neurodegenerative, ischemic/neurovascular, cancer, or peripheral-cell models. Aβ, amyloid-β; BBB, blood–brain barrier; HGF, hepatocyte growth factor; IL-6, interleukin-6; iPSC, induced pluripotent stem cell; OGD, oxygen–glucose deprivation; TGF-β1, transforming growth factor-β1; TNT, tunneling nanotube.

**TABLE 2:** Evidence-informed disease-specific working hypotheses for potential TNT-mediated communication in major central nervous system inflammatory demyelinating disorders.

Disease	Primary pathological entry point	Predicted dominant interacting cell pairs	Lesion microenvironment features potentially favoring TNT-mediated communication	Putative TNT-related roles	Candidate cargoes involved	Current evidence level*	Key unresolved questions
MS	Immune-mediated demyelination with chronic lesion activity	Microglia–astrocytes; astrocytes–oligodendrocyte-lineage cells; glia–axons	Chronic active lesion borders, oxidative stress, mitochondrial dysfunction, persistent gliosis, incomplete remyelination	Pathological burden transfer; metabolic support; maintenance of chronic lesion edge activity; contribution to remyelination failure	Functional/damaged mitochondria, vesicles, lysosomal cargoes, stress-associated intracellular materials	Indirect evidence	Are TNTs enriched at chronic active lesion edges? Do they alter oligodendrocyte survival, axonal integrity, or remyelination outcome?
NMOSD	Primary astrocytopathy with neurovascular involvement	Astrocyte–astrocyte; astrocyte–endothelial/perivascular interfaces; astrocyte–neuron	Astrocytic injury, barrier disruption, complement-associated inflammation, severe tissue stress	Spread of astrocytic stress; compensatory signaling; possible modulation of neurovascular dysfunction	Damaged organelles, inflammatory vesicles, stress-associated cargoes, possibly mitochondria	Low	Do TNTs emerge in astrocyte-rich lesional zones? Are they adaptive or maladaptive in AQP4-IgG-mediated injury?
MOGAD	Antibody-associated inflammatory demyelination with variable recovery	Oligodendrocyte-lineage cells–astrocytes; glia–axons; microglia–glia	Post-inflammatory repair state, glial stress, transient energy imbalance, post-inflammatory repair phase	Repair coordination; metabolic support; influence on recovery efficiency	Mitochondria, vesicles, metabolic cargoes, stress-related materials	Very limited evidence	Are TNTs linked to favorable recovery or persistent repair insufficiency? Which cell pairs are most relevant during lesion resolution?

*Evidence levels refer specifically to the strength of evidence supporting TNT involvement in inflammatory demyelinating disorders, rather than to the established biology of each disease. “Indirect” indicates mechanistic evidence from related CNS or non-demyelinating systems; “Low” indicates limited indirect evidence combined mainly with disease-specific biological plausibility; and “Very limited” indicates predominantly hypothesis-based interpretation with minimal TNT-specific support. Direct disease-specific evidence is currently lacking, and all TNT-related interpretations therefore represent working hypotheses. Established disease features listed in the table, such as chronic active lesion organization in MS or astrocytic injury in AQP4-IgG-positive NMOSD, provide biological context but should not be interpreted as evidence of TNT involvement.

## 3 Biological properties of tunneling nanotubes

TNTs have emerged as candidate structures for direct intercellular communication in the nervous system. Since their first description in 2004, TNTs have been identified in multiple cell types and linked to a growing range of physiological and pathological functions (Dias et al., 2026), including communication among neurons, astrocytes, oligodendrocytes, and microglia (Capobianco et al., 2023; Chakraborty et al., 2023a; Dagar and Subramaniam, 2023; Dias et al., 2026). TNTs are generally described as thin, membrane-enclosed, F-actin-rich protrusions that connect cells over long distances and allow the exchange of intracellular components, ranging from ions and organelles to pathogens and toxic protein aggregates (Chakraborty et al., 2023b; Rustom et al., 2004). Their reported diameter usually ranges from tens to more than 1,000 nm, and their length may reach several times the diameter of the connected cells (Kotarba et al., 2024; Zhou et al., 2024).

TNT formation is a dynamic process involving cell-cell interaction and cytoskeletal remodeling. Two main modes of formation have been proposed. One involves the persistence of membrane bridges after cell separation, whereas the other involves the extension of filopodia-like protrusions that connect with target cells (Chang et al., 2022; Delage et al., 2016). TNTs are therefore not simply passive membrane extensions. They can be actively generated under specific cellular conditions, particularly stress. Several molecular regulators have been implicated in TNT formation. M-Sec promotes membrane nanotube formation through interactions with RalA and the exocyst complex (Hase et al., 2009). LST1 has also been reported to participate in the assembly of molecular machinery required for TNT formation (Schiller et al., 2013). In addition, actin-regulatory proteins such as Eps8 contribute to the distinction between TNTs and filopodia-like structures (Delage et al., 2016).

Through TNT-mediated communication, cells can exchange mitochondria, lysosomes, endosomes, vesicles, protein complexes, RNA-containing structures, and other materials related to stress adaptation or pathological challenge. This mode of transfer may be particularly important under cellular stress, because it enables rapid and spatially restricted exchange between neighboring cells. Experimental studies have shown that hydrogen peroxide exposure or serum depletion induces TNT formation in cultured rat hippocampal astrocytes. Under co-culture conditions, stressed cells show an increased tendency to form TNTs toward unstressed cells (Wang et al., 2011). This process has been linked to p53 activation and downstream EGFR and PI3K/Akt/mTOR signaling (Wang et al., 2011). Indirect evidence from non-demyelinating and peripheral-cell systems suggests that inflammatory and metabolic stress can modulate TNT formation and cargo transfer. TNT-mediated organelle sharing has also been described in communication between stem cells and immune cells, and pro-inflammatory cytokines can induce TNT formation in selected settings (Baghaie et al., 2026). Other conditions, including pathogen infection, oxidative stress, inflammation, and misfolded protein accumulation, have also been associated with TNT formation (Kotarba et al., 2024). However, inflammatory activation does not uniformly increase TNT formation. In macrophages, pro-inflammatory polarization has been reported to inhibit TNT-like processes and intercellular organelle transport (Goodman et al., 2019). In another context, mitochondria are transferred from adipose-derived stem cells to adipose tissue macrophages through TNTs, supporting metabolic adaptability (Chen et al., 2025). These findings indicate that oxidative stress, inflammatory stimulation, and metabolic pressure may regulate TNT formation, but their effects are context-dependent. These observations provide mechanistic insight into stress-regulated TNT biology but should not be interpreted as direct evidence for TNT involvement in inflammatory demyelinating lesions.

A major biological feature of TNTs is their ability to support directional cargo transfer. Recent studies and reviews suggest that TNTs may have dual roles in cargo transport, pathological spread, and neuroprotection in glial cells (Dias et al., 2026; Xu et al., 2026). In the central nervous system, astrocytes are involved in TNT-like networks and may transfer mitochondria, ions, or other cellular cargoes to neurons, thereby supporting neuronal adaptation to stress or injury (Dias et al., 2026). Conversely, TNTs may also act as conduits for pathological agents (Kotarba et al., 2024) and provide a direct route for intercellular transfer of pathological protein aggregates (Dias et al., 2026). TNTs are actin-based cytoplasmic bridges that allow mitochondria to move directly between cells (Zamberlan and Semenzato, 2025). In pathological states associated with mitochondrial dysfunction, such as inflammatory injury, such transfer may help restore mitochondrial function in recipient cells and support cellular survival (Zamberlan and Semenzato, 2025).

TNT-mediated transport should not be regarded as inherently protective. Its effects depend on cell type, cargo identity, transfer direction, and the local microenvironment. Transfer of functional organelles may support stressed cells, whereas damaged organelles, inflammatory signals, or toxic protein aggregates may propagate injury. This duality is particularly relevant to MS and related inflammatory demyelinating disorders, where oxidative stress, mitochondrial dysfunction, inflammation, and energy failure coexist within lesions.

TNTs operate alongside several established CNS communication pathways. Cytokines and other soluble mediators coordinate broader inflammatory responses, whereas connexin-based gap junctions and pannexin channels mainly exchange or release ions and small signaling molecules. Synaptic communication provides rapid signaling between specialized neuronal partners, while extracellular vesicles transport proteins, lipids, nucleic acids, and other cargoes without requiring sustained physical contact. TNTs differ in providing direct membrane-bound connections between defined donor and recipient cells, allowing transfer of relatively large intracellular cargoes, including intact organelles.

TNT formation should not be viewed as a deliberate cellular “choice,” but as a context-dependent stress response. Because maintaining an actin- and membrane-based bridge is likely to carry structural and energetic costs, TNTs would not be expected to replace simpler communication routes under basal conditions. Their potential advantage may emerge when direct donor-recipient coupling enables rapid, spatially restricted transfer of intact mitochondria or other large cargoes, protects cargo from extracellular dilution or degradation, or delivers material directly to a metabolically compromised but viable neighboring cell. Such benefits may become particularly relevant during severe but potentially reversible cellular stress. Whether this proposed cost-benefit relationship operates in inflammatory demyelinating lesions remains unknown.

TNTs and extracellular vesicles share the ability to redistribute biologically active and stress-associated material, and both may have protective or detrimental effects. Their spatial organization, however, differs markedly. Extracellular vesicles are released into the extracellular space and can reach multiple recipient cells, whereas TNTs maintain direct connections between defined cell pairs and support more spatially restricted transfer, including movement of intact intracellular structures. The two systems are therefore more likely to be complementary than mutually exclusive. Extracellular vesicles may distribute signals across a wider cellular field, while TNTs may be more suited to targeted organelle or large-cargo exchange. Because TNTs can also transport vesicular and endolysosomal material, these communication systems may intersect within the same lesion environment.

This distinction is especially important when the biological question concerns donor-recipient identity, transfer direction, or whole-organelle exchange. Extracellular vesicles can demonstrate secreted cargo-mediated effects, but they do not necessarily identify a persistent physical route between a specific cell pair. TNTs may therefore be particularly relevant to mitochondrial transfer, disposal of damaged organelles, or other forms of large-cargo exchange that are not readily explained by diffusion, channel-mediated signaling, or conventional vesicle secretion alone.

It remains unclear whether TNTs ever dominate over other communication pathways in inflammatory demyelinating lesions. Their relative contribution may instead increase when metabolically or oxidatively stressed but viable cells require rapid, localized transfer of large intracellular cargoes. Such conditions may occur at lesion borders, within stressed glial networks, or at neurovascular interfaces. By contrast, cytokines, extracellular vesicles, and channel-mediated signaling may remain more important for broader inflammatory or small-molecule communication. These pathways are therefore likely to operate in parallel, with their relative contribution varying by cell type, lesion stage, tissue architecture, and cargo. Functional redundancy should also be considered, as it is unknown whether other communication routes can compensate when TNT-mediated exchange is reduced.

Methodological limitations remain substantial. TNTs lack highly specific molecular markers (Capobianco et al., 2023), and their identification therefore depends on convergent morphological and functional evidence, including cytoskeletal composition, live-cell imaging, and cargo tracking. Distinguishing TNTs from filopodia, cytonemes, retraction fibers, and other membrane protrusions is particularly difficult in complex tissues. Their small diameter and fragility also make them vulnerable to disruption during fixation and sample preparation, increasing the risk of under-detection or misclassification.

Clarifying the role of TNTs in inflammatory demyelinating disease will therefore require several steps. TNTs must first be identified using stringent structural and functional criteria. Disease-relevant stressors, including inflammation, oxidative stress, metabolic dysfunction, and barrier stress, should then be examined as regulators of TNT formation. Finally, TNT function should be interpreted in relation to the participating cell types, lesion stage, transfer direction, and cargo identity, rather than inferred from the presence of TNT-like structures alone.

## 4 An integrative model linking cellular stress, TNT formation, and lesion evolution

Direct evidence for TNT-mediated communication in inflammatory demyelinating disorders remains scarce, and functional cargo transfer has not been convincingly demonstrated *in situ* in human MS, NMOSD, or MOGAD lesions. The model proposed here should therefore be regarded as a working hypothesis integrating disease biology with mechanistic evidence from other experimental systems.

Within this framework, TNT formation is viewed not as an independent pathological pathway but as a stress-responsive output of oxidative imbalance, mitochondrial dysfunction, cytoskeletal remodeling, and metabolic stress. By providing direct intercellular cargo exchange, TNTs could contribute to pathological burden transfer, metabolic support through mitochondrial or other intracellular resource exchange, and repair coordination among glial, neural, and vascular-associated cells. TNT-mediated communication may therefore link persistent lesion activity with incomplete remyelination. This possibility must be interpreted in a disease-specific manner. NMOSD and MOGAD are related to MS within the broader spectrum of inflammatory central nervous system demyelination, but they have distinct pathological mechanisms (Uzawa et al., 2024). AQP4-IgG-positive NMOSD is characterized primarily by astrocytic injury related to aquaporin-4 (Xu et al., 2025). MOGAD is a distinct central nervous system demyelinating disorder targeting a myelin/oligodendrocyte surface antigen. These diseases differ in complement activation, lesion histology, oligodendrocyte preservation, tissue-damage pattern, repair potential, and neurovascular involvement. Common pathological conditions, including inflammation, oxidative stress, metabolic disturbance, barrier stress, and limited repair, may create environments favorable to TNT-mediated communication. However, the relevant cell-cell interactions and lesion stages are likely to differ among these disorders.

At the lesion scale, TNTs may be viewed as one component of a broader communication network rather than as isolated pairwise bridges. Their biological significance may depend on how TNT-mediated connections redistribute metabolic support, stress signals, or pathological cargo across spatially organized cellular states.

Mechanistically, TNT-mediated communication can be considered in terms of four linked components: the sender cell, the transferred cargo, the recipient cell, and the resulting response. The same cargo may produce different effects depending on the metabolic and inflammatory state of the recipient, while transfer may be unidirectional or bidirectional and may change over time. Important unresolved questions include how cargo is selected, how transfer direction is determined, how recipient cells respond, and how TNT-mediated communication is terminated.

This model should also account for glial heterogeneity. Single-cell and spatial studies show that microglia, astrocytes, and oligodendrocyte-lineage cells adopt distinct states according to lesion location and stage. TNT formation and cargo transfer may therefore depend not only on cell identity but also on local activation state, metabolic condition, and spatial position. For example, astrocytes at active lesion rims may differ substantially from those in inactive lesion cores or normal-appearing white matter, and similar state dependence may apply to microglia and oligodendrocyte-lineage cells.

Within this framework, MS, NMOSD, and MOGAD need not involve separate TNT mechanisms. Their distinct lesion biology may instead determine the source of cellular stress, the participating cell states, the cargoes exchanged, and whether TNT-mediated communication favors tissue protection or lesion persistence. The following sections consider how this shared model may operate in lesion propagation, remyelination, and the distinct pathological settings of these disorders.

## 5 TNTs in lesion propagation and local tissue stress

Inflammatory demyelinating lesions are dynamic rather than static structures. Their progression reflects not only the initiating immune insult but also sustained interactions within the lesion microenvironment. In MS, chronic active lesions are not inert scars. They are pathological units with persistent marginal activity, characterized by enrichment of activated microglia and macrophages at lesion edges, together with ongoing demyelination, axonal injury, and glial activation (Bagnato et al., 2024; Boutitah-Benyaich et al., 2025; Dal-Bianco et al., 2024). Even when peripheral immune activity is partly suppressed, local tissue conditions may remain unfavorable because of persistent glial activation, oxidative stress, and metabolic disequilibrium. This suggests that lesion expansion may be maintained, at least in part, by pathological interactions among resident central nervous system cells.

This model is particularly relevant to lesion borders, where partially injured but still viable cells coexist with activated glia and vulnerable axons. In these regions, TNT-mediated transfer could propagate stress-related signals and help maintain chronic lesion activity. Such a mechanism may help explain why some lesions remain biologically active even in the absence of prominent new immune-cell infiltration. At lesion borders, TNT-mediated communication could either propagate stress-associated cargo or provide metabolic support, with its net effect likely determined by lesion stage, participating cell types, and cargo identity.

## 6 TNTs and myelin repair: linking metabolic support to remyelination failure

TNTs may be particularly relevant to myelin repair. Successful remyelination requires OPC recruitment and differentiation within a lesion environment that supports metabolism, limits chronic inflammation, clears myelin debris, and preserves axon–glia interactions. In inflammatory demyelinating disease, repair may be initiated but fail to progress sufficiently to restore tissue integrity.

After demyelination, axons become increasingly dependent on local metabolic support, while oligodendrocyte-lineage cells require adequate mitochondrial function and lipid synthesis for remyelination. TNT-mediated transfer of functional or damaged mitochondria and other stress-related cargoes could therefore influence whether lesions become repair-permissive or repair-inhibitory. More broadly, metabolic recovery also depends on glucose utilization, axon-glia metabolic coupling, and lipid and cholesterol synthesis required for myelin production. Iron handling and associated oxidative stress represent another potentially important metabolic component, although their relationship to TNT-mediated communication remains largely unexplored. TNTs should therefore be considered within the broader metabolic network that shapes remyelination rather than solely as a pathway for mitochondrial transfer. The blood-brain barrier and neurovascular unit provide another entry point for considering TNTs in inflammatory demyelinating disorders. Acute lesion formation is often associated with blood-brain barrier dysfunction, reflecting altered interactions among endothelial cells, perivascular cells, astrocytes, and the basement membrane. Because direct evidence from inflammatory demyelinating lesions is lacking, findings from cerebrovascular models are considered here only as indirect mechanistic evidence. In ischemia/reperfusion models, TNTs facilitate communication between peripheral cells and blood-brain barrier-associated cells, and peripheral cells can transfer mitochondria to compromised astrocytes through TNTs, thereby reducing apoptosis and contributing to blood-brain barrier (BBB) repair (Pisani et al., 2022).

Indirect evidence from neurodegenerative and neuronal-stress models further illustrates the potential capacity of TNTs to regulate glial stress handling and organelle exchange. In other central nervous system disease models, astrocyte-microglia interactions through nanotube-like structures have been associated with enhanced degradation of α-synuclein and amyloid-β aggregates (Rostami et al., 2021). Under neuronal stress, microglia can use TNTs to remove aggregates from neurons and transfer healthy mitochondria, thereby reducing oxidative stress and supporting neuronal recovery (Scheiblich et al., 2024). Although these findings demonstrate relevant TNT functions in the CNS, they do not establish that the same mechanisms operate in inflammatory demyelinating lesions.

Effective remyelination requires coordinated responses from microglia, astrocytes, OPCs, mature oligodendrocytes, and axons. TNT-mediated communication may influence this coordination by shaping how stress signals, metabolic resources, and intracellular cargoes are distributed within the lesion. As a result, TNTs could affect not only oligodendroglial survival but also the balance between repair-permissive and repair-inhibitory microenvironments.

## 7 Disease-specific considerations in MS, NMOSD, and MOGAD

### 7.1 Shared pathological conditions that may favor TNT-mediated communication

MS, NMOSD, and MOGAD share several lesion features that could favor TNT-mediated communication, including inflammation, oxidative stress, mitochondrial dysfunction, metabolic imbalance, neurovascular stress, and incomplete repair. These conditions overlap with stressors linked to TNT formation in other experimental systems, providing a rationale for considering TNTs in inflammatory demyelination.

However, shared pathology does not imply equivalent TNT function. Its significance is likely to depend on the primary cellular target, the participating cell populations, and the dominant lesion requirement, such as stress propagation, metabolic support, barrier repair, or remyelination. The disease-specific hypotheses below therefore integrate limited disease-relevant observations with indirect mechanistic evidence rather than established TNT mechanisms. Each disorder is considered according to four dimensions: conditions favoring TNT formation, likely participating cells, plausible cargoes, and the lesion stage at which TNT-mediated communication may be most relevant.

### 7.2 MS: a working hypothesis centered on chronic lesion edge activity and remyelination failure

Our working hypothesis for MS is that TNT-mediated communication may be most relevant at chronic active lesion edges, where distinct lesion-associated microglial and astrocytic states coexist with metabolically stressed oligodendrocyte-lineage cells and vulnerable axons. This hypothesis is biologically supported by several features of chronic MS lesions, including persistent glial activation, oxidative and mitochondrial stress, impaired oligodendrocyte metabolism, and increased dependence of demyelinated axons on local metabolic support. TNT formation and cargo handling are unlikely to be uniform across these glial populations and may instead vary with their inflammatory, repair-associated, metabolic, and spatial states within the lesion. The chronic inflammatory environment in MS not only drives tissue injury but also restricts repair. Remyelination failure has been linked to glucose and lipid metabolic imbalance, mitochondrial dysfunction, and altered metabolic support. Oligodendrocyte structural damage and functional loss are central to MS pathology. Oligodendrocytes are highly dependent on mitochondrial and lipid metabolism, and lipid synthesis and metabolic reprogramming are closely related to myelination and regeneration (Sun et al., 2026). After demyelination, axons become more dependent on local energy support, and the metabolic support provided by glial cells can influence axonal outcome (Looser et al., 2024).

Chronic active MS lesion rims may provide a permissive environment for TNT-mediated communication because persistent microglial and astrocytic activation coexists with oxidative stress, mitochondrial dysfunction, and increased metabolic demand in partially injured but viable cells. Plausible interactions include microglia–astrocyte, astrocyte–oligodendrocyte-lineage, and glia–axon connections within these spatially restricted niches. Functional outcomes would depend on cargo identity: transfer of functional mitochondria or other metabolic components could support stressed oligodendrocytes or demyelinated axons, whereas damaged mitochondria, lysosomal or vesicular material, and other stress-associated cargoes could reinforce oxidative injury and glial activation. TNT-mediated communication may therefore be less relevant during the initial immune-driven phase, but more important at chronic active lesion edges and during later repair, where its effects may range from metabolic support to remyelination failure.

Single-cell and spatial studies further indicate substantial heterogeneity among glial populations at MS lesion edges. Chronic active lesions contain distinct inflammatory microglial and astrocytic states, while lesion-rim niches show locally organized myeloid, astrocytic, oligodendroglial, and endothelial programs. Oligodendrocyte populations also differ in vulnerability and responses to inflammatory stress. TNT-mediated communication may therefore preferentially arise among specific stress-associated cell states rather than uniformly across glial populations.

On this basis, the potential significance of TNTs in MS may not be limited to inflammatory signal transfer. TNTs may also provide a direct cellular connection involved in metabolic compensation, organelle transfer, and rescue of damaged cells (Henrich and Scheiblich, 2026; Xu et al., 2026). At chronic lesion edges, TNT-mediated communication could facilitate pathological burden transfer, sustain glial activation, redistribute metabolic resources, or interfere with complete repair. TNTs are therefore particularly relevant to two major problems in MS pathology: chronic lesion expansion and remyelination failure.

TNT involvement in MS may be more relevant during chronic lesion evolution than during the initial acute immune attack. In chronic lesions, resident glial networks may sustain a low-grade, self-reinforcing pathological state even when overt inflammation is partly controlled. TNT-mediated communication could either amplify this state by spreading stress-related cargoes or counteract injury through metabolic support, potentially contributing to persistent lesion activity and repair resistance.

The role of TNTs is also likely to change across lesion stages. During acute inflammation, immune-cell infiltration and soluble signaling may predominate, whereas direct communication between stressed resident cells may become more important at chronic active or slowly expanding lesion edges. During resolution and remyelination, TNT-mediated metabolic support could favor recovery, while maladaptive cargo transfer could contribute to incomplete repair. Progressive MS may therefore represent a particularly relevant setting in which to investigate sustained TNT-mediated communication.

### 7.3 NMOSD: a working hypothesis centered on astrocytic and neurovascular stress

Our working hypothesis for AQP4-IgG-positive NMOSD is that TNT-mediated communication, if present, would be most relevant within astrocyte-centered and neurovascular networks rather than oligodendrocyte-centered repair pathways. This is based on the primary astrocytopathy of NMOSD, in which astrocytic injury is associated with blood–brain barrier disruption, complement-mediated inflammation, metabolic stress, and secondary damage to surrounding neural cells. As key components of both glial metabolic networks and the neurovascular unit, stressed astrocytes could provide conditions favoring TNT-mediated transfer of mitochondria or other stress-associated cargoes.

Unlike MS, however, state- and spatially resolved characterization of astrocytic populations in human NMOSD lesions remains limited, making it premature to assign TNT-related functions to specific astrocytic states.

TNT-mediated communication could either propagate pathological stress or support compensatory responses, depending on the condition of the astrocytic network. It may spread injury-related cargoes across connected cells or help surviving cells restore local homeostasis. Its significance in NMOSD should therefore be considered in relation to astrocytic network disruption, neurovascular stress, blood–brain barrier integrity, and secondary effects on oligodendrocytes and axons.

### 7.4 MOGAD: a highly tentative working hypothesis for post-inflammatory repair

The proposed role of TNTs in MOGAD is the most tentative of the three disease-specific hypotheses because direct disease-relevant evidence is essentially lacking. Unlike MS, where chronic active lesion edges provide a plausible setting for persistent local glial communication, or AQP4-IgG-positive NMOSD, where primary astrocytic injury offers a defined cellular entry point, MOGAD lacks an equally well-established TNT-relevant lesion niche.

The mechanistic context in MOGAD may therefore differ from that in MS or NMOSD. Acute inflammatory demyelination and injury to myelin-associated structures may generate metabolic and oxidative stress, but TNT-mediated communication may be more relevant during lesion resolution and remyelination than during the acute attack. At this stage, oligodendrocyte-lineage cells, astrocytes, microglia, and demyelinated axons must coordinate metabolic recovery and tissue repair. If present, TNT-mediated transfer would most plausibly involve oligodendrocyte-lineage-astrocyte or glia-axon interactions. Functional mitochondria and other metabolic or vesicular cargoes could support energy supply and recovery, whereas damaged organelles could prolong metabolic dysfunction and impair repair. However, neither the relevant cell pairs nor the transferred cargoes have been demonstrated directly in MOGAD.

We therefore propose cautiously that TNT-mediated communication in MOGAD, if present, may be more important during post-inflammatory repair than during chronic lesion propagation. Because MOGAD primarily targets myelin/oligodendrocyte-associated structures and recovery after acute inflammation is variable, coordinated metabolic and stress responses among glial cells and axons are likely to be important. TNT-mediated exchange of mitochondria or other metabolic cargoes could represent one such repair mechanism, but this remains a testable hypothesis rather than an established feature of MOGAD.

### 7.5 Current interpretation

Taken together, these disease-specific interpretations should be viewed as working hypotheses. The underlying rationale is strongest for chronic lesion-associated glial communication in MS, less direct for astrocyte-centered pathology in NMOSD, and most speculative for MOGAD. This hierarchy of plausibility may help guide future disease- and lesion-specific studies. These disease-specific cellular contexts, proposed TNT functions, candidate cargoes, and corresponding evidence levels are summarized in Table 2. An integrated working model of these proposed TNT-mediated interactions is shown in Figure 1.

**FIGURE 1:**
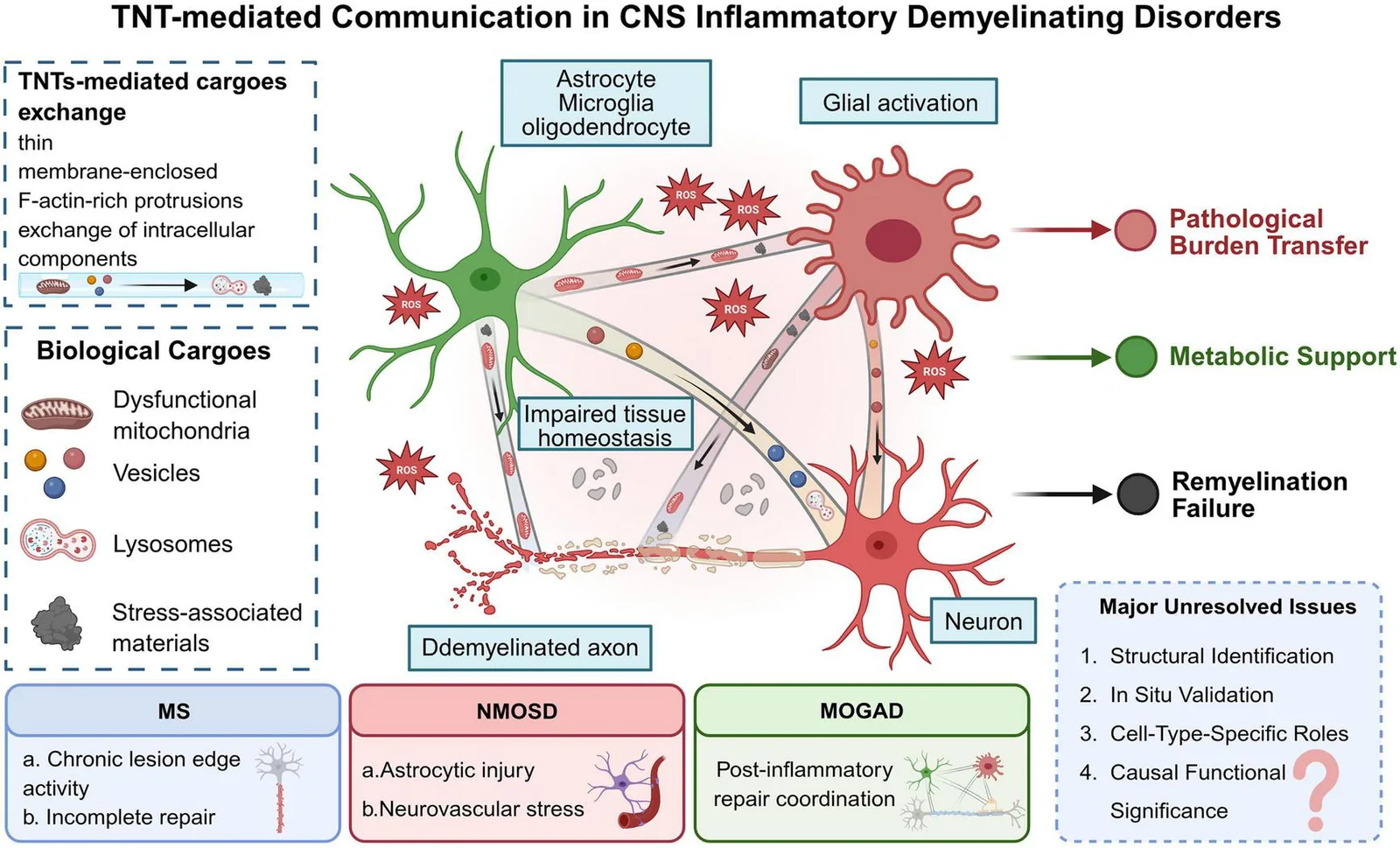
Working model of potential TNT-mediated communication in central nervous system inflammatory demyelinating disorders. Created using BioRender.com.

## 8 Main limitations of current evidence and future directions

Despite their conceptual relevance, TNTs remain difficult to integrate into inflammatory demyelinating disease because several fundamental questions are unresolved. The key issue is not simply whether TNT-like structures can be observed experimentally, but whether they can be rigorously identified within lesions, assigned to specific cell types, linked to defined cargo transfer, and shown to affect pathological outcomes. Aging may further modify TNT biology through changes in glial state, mitochondrial quality, cytoskeletal plasticity, and repair capacity, which may be particularly relevant to progressive MS and age-related remyelination failure.

### 8.1 Difficulty in structural identification

A major technical challenge is the lack of a specific molecular marker for TNTs. Reliable identification therefore requires convergent structural and functional evidence rather than morphology alone. Ideally, this should include F-actin enrichment, membrane and, where applicable, cytoplasmic continuity, three-dimensional imaging confirming intercellular connectivity and lack of substrate attachment, live-cell visualization, and direct cargo tracking. These criteria are essential for distinguishing TNTs from filopodia, cytonemes, retraction fibers, and other membrane extensions. Identification is especially difficult in demyelinating lesions because of complex tissue architecture, abundant glial processes, and the fragility of TNTs during fixation and sample preparation.

### 8.2 Lack of *in situ* validation in lesions

Direct *in situ* validation in disease-relevant tissue remains limited. In MS, NMOSD, and MOGAD, it remains unclear whether TNT-like structures are consistently present within lesions, which cell types form them, and whether their frequency or properties vary with lesion stage, severity, or repair status. Most evidence comes from *in vitro* systems, with little direct confirmation in animal models or human lesions. Demonstrating TNTs and associated cargo transfer *in situ* is therefore necessary before stronger disease-specific conclusions can be drawn.

### 8.3 Imbalance of evidence across cell types

Evidence is also uneven across cell populations. Most studies have focused on neurons, astrocytes, microglia, and immune-related cells, whereas oligodendrocytes and oligodendrocyte-lineage cells remain underrepresented. This is an important gap given their central role in myelin injury and remyelination. Future work should therefore examine TNT-mediated communication within the oligodendrocyte lineage, particularly in relation to metabolic support, stress adaptation, and repair.

### 8.4 Limited causal evidence and functional interpretation

As summarized in Table 1, most mechanistic evidence derives from CNS stress, neurodegenerative, ischemic, or non-CNS models rather than inflammatory demyelinating lesions. Causal evidence is therefore still limited. Key questions include which cargoes are transferred, in which direction, under what conditions, and with what functional consequences. Functional mitochondria, damaged organelles, inflammatory vesicles, and myelin-related degradation products could have markedly different effects.

Future studies should combine manipulation of TNT formation with cargo tracking and functional outcomes, including mitochondrial activity, glial activation, axonal integrity, remyelination, and neurological recovery. Therapeutically, TNTs are unlikely to represent a uniformly beneficial or harmful target. Inhibition might reduce pathological cargo spread but impair metabolic rescue, whereas enhancing TNT formation could support stressed cells while facilitating maladaptive transfer. Any intervention will therefore need to account for cell state, cargo identity, transfer direction, and lesion stage.

### 8.5 Experimental roadmap for establishing TNT function in demyelinating disease

A stepwise strategy is needed to establish the causal importance of TNT-mediated communication. First, live-cell imaging, three-dimensional microscopy, and direct cargo tracking should verify TNT formation, transfer direction, and organelle exchange under disease-relevant inflammatory, oxidative, or metabolic stress.

Second, the relevant cellular states and lesion niches should be defined. Single-cell and spatial transcriptomic approaches can identify candidate cell states, but should be combined with spatial proteomics, imaging mass cytometry, multiplex imaging, and high-resolution three-dimensional microscopy because spatial omics alone cannot demonstrate TNT formation or cargo transfer. Patient-derived iPSC co-cultures, organoids, and glial–neurovascular assembloids may provide useful intermediate models between conventional culture and human tissue.

Third, biological relevance requires quantitative assessment. Studies should measure TNT frequency and density, the proportion of cells participating in TNT networks, and the amount and rate of cargo transfer. These parameters should also be compared with extracellular vesicles, soluble mediators, and channel-mediated signaling, while considering the energetic and structural costs of TNT formation.

Finally, causal studies should test whether altering TNT formation changes lesion-relevant outcomes. Genetic or pharmacological manipulation of candidate TNT regulators should be coupled with direct assessment of TNT formation, cargo transfer, mitochondrial function, oxidative stress, glial activation, axonal integrity, and remyelination. Because available TNT-regulatory pathways are not highly specific, rescue experiments will be important for separating TNT-dependent effects from broader cytoskeletal changes.

Clinical translation remains challenging because no validated assay directly measures TNT activity in patients. High-resolution tissue imaging remains the most direct approach, whereas CSF or blood measurements would represent indirect surrogates unless TNT-associated cargoes or regulatory signatures can be validated. Such measurable correlates will be needed before TNT biology can be linked reliably to disease activity or treatment response.

## 9 Conclusions and future directions

TNTs provide a plausible framework for direct, stress-responsive intercellular communication in inflammatory demyelinating lesions, linking pathological cargo transfer, metabolic support, and repair. Their relevance is likely to depend on disease and lesion context, with chronic lesion activity in MS, astrocytic and neurovascular stress in NMOSD, and post-inflammatory repair in MOGAD representing distinct working hypotheses. Establishing disease relevance will require rigorous *in situ* identification, cell-specific cargo tracking, and causal studies in demyelinating models and human tissues.

Established features of inflammatory demyelinating lesions include inflammation, oxidative stress, mitochondrial dysfunction, glial activation, neurovascular stress, and impaired repair. These provide the biological context for the proposed TNT-mediated pathways shown in the figure. Solid elements indicate established disease-associated observations, whereas dashed elements denote proposed TNT-mediated relationships and disease-specific working hypotheses. The model suggests that TNTs may contribute to pathological cargo transfer, metabolic support, and altered repair coordination. Potential disease-specific roles include chronic lesion edge activity and remyelination failure in MS, astrocytic injury and neurovascular stress in NMOSD, and post-inflammatory repair in MOGAD. These pathways remain hypothetical and require direct *in situ* and causal validation.
